# Faecalibacterium taiwanense sp. nov., isolated from human faeces

**DOI:** 10.1099/ijsem.0.006413

**Published:** 2024-06-07

**Authors:** Jong-Shian Liou, Wei-Ling Zhang, Li-Wen Hsu, Chih-Chieh Chen, Yu-Ting Wang, Koji Mori, Kohei Hidaka, Moriyuki Hamada, Lina Huang, Koichi Watanabe, Chien-Hsun Huang

**Affiliations:** 1 Bioresource Collection and Research Center, Food Industry Research and Development Institute, 331 Shih-Pin Rd, Hsinchu 30062, Taiwan, ROC; 2 Institute of Medical Science and Technology, National Sun Yat-sen University, Kaohsiung 80424, Taiwan, ROC; 3 Rapid Screening Research Center for Toxicology and Biomedicine, National Sun Yat-sen University, Kaohsiung 80424, Taiwan, ROC; 4 Division of Research and Analysis, Food and Drug Administration, Ministry of Health and Welfare, Taipei 11561, Taiwan, ROC; 5 Biological Resource Center, National Institute of Technology and Evaluation (NBRC), 2-5-8 Kazusakamatari, Kisarazu, Chiba 292-0818, Japan; 6 Department of Animal Science and Technology, National Taiwan University, No. 50, Lane 155, Sec 3, Keelung Rd., Taipei 10673, Taiwan, ROC

**Keywords:** *Faecalibacterium prausnitzii* group, *Faecalibacterium taiwanense*, human faeces

## Abstract

Two Gram-stain-negative, straight rods, non-motile, asporogenous, catalase-negative and obligately anaerobic butyrate-producing strains, HLW78^T^ and CYL33, were isolated from faecal samples of two healthy Taiwanese adults. Phylogenetic analyses of 16S rRNA and DNA mismatch repair protein MutL (*mutL*) gene sequences revealed that these two novel strains belonged to the genus *Faecalibacterium*. On the basis of 16S rRNA and *mutL* gene sequence similarities, the type strains *Faecalibacterium butyricigenerans* AF52-21^T^(98.3–98.1 % and 79.0–79.5 % similarity), *Faecalibacterium duncaniae* A2-165^T^(97.8–97.9 % and 70.9–80.1 %), *Faecalibacterium hattorii* APC922/41-1^T^(97.1–97.3 % and 80.3–80.5 %), *Faecalibacterium longum* CM04-06^T^(97.8–98.0% and 78.3 %) and *Faecalibacterium prausnitzii* ATCC 27768^T^(97.3–97.4 % and 82.7–82.9 %) were the closest neighbours to the novel strains HLW78^T^ and CYL33. Strains HLW78^T^ and CYL33 had 99.4 % both the 16S rRNA and *mutL* gene sequence similarities, 97.9 % average nucleotide identity (ANI), 96.3 % average amino acid identity (AAI), and 80.5 % digital DNA–DNA hybridization (dDDH) values, indicating that these two strains are members of the same species. Phylogenomic tree analysis indicated that strains HLW78^T^ and CYL33 formed an independent robust cluster together with *F. prausnitzii* ATCC 27768^T^. The ANI, AAI and dDDH values between strain HLW78^T^ and its closest neighbours were below the species delineation thresholds of 77.6–85.1 %, 71.4–85.2 % and 28.3–30.9 %, respectively. The two novel strains could be differentiated from the type strains of their closest *Faecalibacterium* species based on their cellular fatty acid compositions, which contained C_18 : 1_* ω*7*c* and lacked C_15 : 0_ and C_17 : 1_* ω*6*c*, respectively. Phenotypic, chemotaxonomic and genotypic test results demonstrated that the two novel strains HLW78^T^ and CYL33 represented a single, novel species within the genus *Faecalibacterium*, for which the name *Faecalibacterium taiwanense* sp. nov. is proposed. The type strain is HLW78^T^ (=BCRC 81397^T^=NBRC 116372^T^).

## Introduction

*Faecalibacterium* species are butyric acid-producing bacteria that play important roles in maintaining gut health. *Faecalibacterium* is a prevalent bacterial group in the healthy human gut and represents more than 5 % of the total bacterial population [1]. The levels of *Faecalibacterium* secies are frequently reduced in diseases linked to gut inflammation, such as inflammatory bowel disease and colorectal cancer [2,6]. Recent studies have demonstrated that *Faecalibacterium* species can effectively reduce inflammation in mouse models of colitis by secreting bioactive compounds such as butyrate, MAM proteins and salicylic acid, which enhance the intestinal barrier [7,16]. The increasing interest in using these gut bacteria as potential candidates for developing next-generation probiotics or live biotherapeutic products shows great promise.

Currently, the genus *Faecalibacterium* is a member of the family *Ruminococcaceae* in the order *Clostridiales*, and consists of six validated species: *Faecalibacterium butyricigenerans*, *Faecalibacterium duncaniae*, *Faecalibacterium gallinarum*, *Faecalibacterium hattorii*, *Faecalibacterium longum* and *Faecalibacterium prausnitzii* (https://lpsn.dsmz.de/genus/faecalibacterium, accessed on 22 May 2024). Most of these species were published validly in recent years, except for *F. prausnitzii* [17,18]. A whole metagenome analysis revealed that a total of 22 different *Faecalibacterium*-like species were present in human, monkey, dog, pig and chicken guts. Among these species, 12 are commonly prevalent in the human gut [19]. As *Faecalibacterium* species are extremely oxygen-sensitive bacteria and difficult to culture in ordinary laboratories [20], more than half of the *Faecalibacterium* population in the human gut remains uncultivated [19].

The isolation, identification and characterization of nonculturable bacteria, especially keystone species, are crucial for enhancing our understanding of the physiological impact of the gut microbiome on the host. During a study focusing on isolating novel *Faecalibacterium* species from the human faeces using a culturomics approach, two isolated strains (HLW78^T^ and CYL33) were not classified as any recognized species of the genus *Faecalibacterium* using matrix-assisted laser desorption ionization time-of-flight mass spectrometry (MALDI-TOF MS) analysis. To delineate the concise taxonomy of strains HLW78^T^ and CYL33, in this study, a polyphasic taxonomic approach was used.

## Isolation and ecology

This study was approved by the Ethical Committee of the Chung Shan Medical University Hospital (IRB Number: CS1-20043). Informed consent was obtained from all the subjects before the study. The freshly voided faecal samples of healthy Taiwanese adults were immediately placed in an anaerobic chamber (Coy Laboratory; atmosphere 80 : 5 : 15 N_2_:CO_2_:H_2_). The samples were homogenized, and serial dilutions were plated onto M2GSC agar [21] and then incubated anaerobically at 37 °C for 48 h. All of the isolates were subjected to strain dereplication using an MALDI Microflex LT mass spectrometer (Bruker Daltonics), as previously described [22]. Strains HLW78^T^ and CYL33 yielded a log score of less than 1.7 and were not reliably identified (data not shown). *F. prausnitzii* ATCC 27768^T^ (=BCRC 81047^T^) was used as a reference strain for further characterization. The isolates and reference strain were routinely cultured on brain heart infusion supplemented (BHIS) agar at 37 °C and preserved in 10 % glycerol at −80 °C. BHIS medium contained 37 g l^−1^ BHI, 3 g l^−1^ yeast extract, 3 g l^−1^ l-cysteine, 3 g l^−1^ sodium acetate, 1 mg l^−1^ resazurin and 20 g l^−1^agar.

## 16S rRNA and *mutL* gene phylogenies

**Fig. 1.**
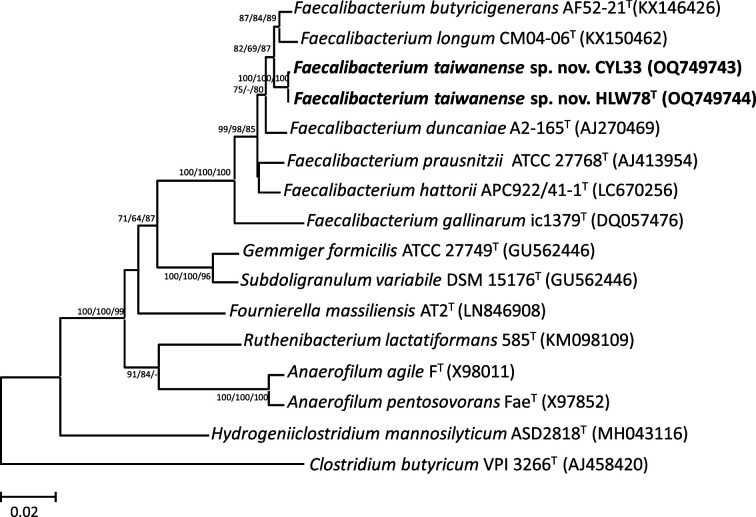
Phylogenetic tree based on 16S rRNA gene sequences showing the relationship of *Faecalibacterium taiwanense* sp. nov. with the type strains of closely related species. The tree was reconstructed by the neighbour-joining, minimum-evolution and maximum-likelihood methods based on a comparison of approximately 1373 bp, and *Clostridium butyricum* VPI 3266^T^ was used as an outgroup. Bootstrap values (>60 %) based on 1000 replicates are shown at branch nodes. Bar, 0.02 sequence divergence.

**Fig. 2.**
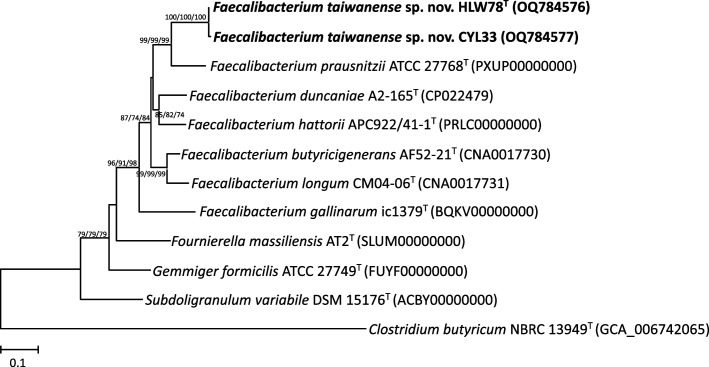
Phylogenetic tree based on *mutL* gene sequences showing the relationship of *Faecalibacterium taiwanense* sp. nov. with the type strains of closely related species. The tree was reconstructed by the neighbour-joining, minimum-evolution and maximum-likelihood methods based on a comparison of approximately 500 bp, and *Clostridium butyricum* NBRC 13949^T^ was used as an outgroup. Bootstrap values (>70 %) based on 1000 replicates are shown at branch nodes. Bar, 0.1 sequence divergence.

A DNeasy kit (Qiagen) was used to extract and purify the bacterial genomic DNA. Amplification and sequencing of the 16S rRNA and *mutL* genes were conducted as previously described [22,23]. The degenerate PCR primers for the *F. prausnitzii* group, FPGmutL-F (5′-TTYATCCGCCAYGCCACCAG-3′) and FPGmutL-R (5′-RCTKCGGCTRGCWCGGCAGC-3′), were designed by comparison with the DNA mismatch repair protein MutL (*mutL*) gene sequences from the genomes among the *Faecalibacterium* species. blast analysis was performed using the NCBI database (http://www.ncbi.nlm.nih.gov/BLAST/). Multiple sequence alignments were performed using Clustal X (version 2.1) software [24]. Phylogenetic trees were reconstructed using mega (version 11) software [25]. Approximately 1373 bp of the 16S rRNA gene sequences were used to reconstruct phylogenetic trees from the isolated strains (HLW78^T^ and CYL33) and related type strains via the neighbour-joining (NJ) [26], minimum-evolution (ME) [27] and the maximum-likelihood (ML) methods [28] with the Kimura two-parameter model. The statistical reliability of the trees was evaluated through bootstrapping analysis of 1000 replicates [29]. The tree topology was also confirmed using the maximum-parsimony (MP) method [30]. On the basis of the 16S rRNA gene sequence similarity, strains *F. butyricigenerans* AF52-21^T^ (98.3 % similarity), *F. duncaniae* A2-165^T^ (97.9 %), *F. hattorii* APC922/41-1^T^ (97.3 %), *F. longum* CM04-06^T^ (98.0 %) and *F. prausnitzii* ATCC 27768^T^ (97.4 %) were the closest neighbours to the novel strain HLW78^T^. Based on the phylogenetic analysis, it was observed that strains HLW78^T^ and CYL33 belonged to an independent cluster within the genus *Faecalibacterium* and were closely related to *F. butyricigenerans* AF52-21^T^ and *F. longum* CM04-06^T^ in the NJ, ME, ML and MP trees, respectively (Figs 1 and S1, available in the online Supplementary Material). The *mutL* gene has been identified as a crucial taxonomic marker with deep-level phylogeny for phylogenetically and phenotypically closely related species [22,31] . The similarity levels of the *mutL* gene sequences between strain HLW78^T^ and its closest neighbours ranged from 79.5 to 82.8 %. The phylogenetic trees reconstructed using the NJ, ME and ML methods based on the *mutL* gene sequences showed that strains HLW78^T^ and CYL33 formed an independent cluster that was clearly separate from those of other closely related species, and the nodes were supported by high bootstrap values (100 %), indicating that these two strains could be novel species within the genus *Faecalibacterium* (Fig. 2). In addition, two novel strains HLW78^T^ and CYL33 exhibited 99.4 % both the 16S rRNA and *mutL* gene sequence similarities, suggesting that these strains could be classified as the same species.

## Genome features

Whole-genome sequencing of HLW78^T^ and CYL33 was performed at Biotools Co., Ltd. (New Taipei City, Taiwan) on the Nanopore PromethION sequencing platform. The raw reads were assembled by Flye (version 2.8.3) [32]. The primary contigs were then polished with model correction by Medaka (version 1.2.3) [33]. Additionally, the contigs were corrected using homologous sequences extracted from closely related genomes with Homopolish (version 0.3.4) [34]. The genome’s completeness and contamination levels were evaluated using CheckM (version 1.2.2) [35]. Genome annotation was carried out using the dfast annotation pipeline (version 1.2.0) [36]. The OrthoVenn2 webserver was used for pangenome analysis [37]. The eggNOG 4.5 database and carbohydrate-active enzyme (CAZy) database were used for functional assignment [38]. The primary metabolite gene clusters were predicted using gutSMASH (version 1.0) software [39]. The antimicrobial resistance, virulence factor and pathogenic genes were searched in the genomes of strains HLW78^T^ and CYL33 based on the Comprehensive Antibiotic Resistance Database Variants version 4.0.0 [40], ResFinder version 4.0 [41], AMRFinderPlus version 3.10.42 [42], the Virulence Factor Database (June 2022 release) [43], CGE VirulenceFinder 2.0.3 [44] and the Pathogen Host Interactions database version 4.14 [45] through the ProbioMinServer [46]. PlasmidFinder version 2.0.1 and PlasmidHunter version 1.0 were used for the detection of plasmids [47,48]. The genome sequences of the reference strains were downloaded from the NCBI database. The phylogenomic trees were reconstructed using the Orthologous ANI Tool based on average nucleotide identity (ANI) values [49], the Type (Strain) Genome Server (https://tygs.dsmz.de/ [50]) and the Up-to-date Bacterial Core Gene (UBCG) pipeline (http://leb.snu.ac.kr/ubcg2 [51]). Moreover, a heatmap, an NJ dendrogram and hierarchical clustering based on the gene content (presence or absence) and ANI values were also generated using the ComplexHeatmap R package [52]. The ANI, digital DNA–DNA hybridization (dDDH) and amino acid identity (AAI) values were calculated as described previously [49, 53,54].

**Fig. 3.**
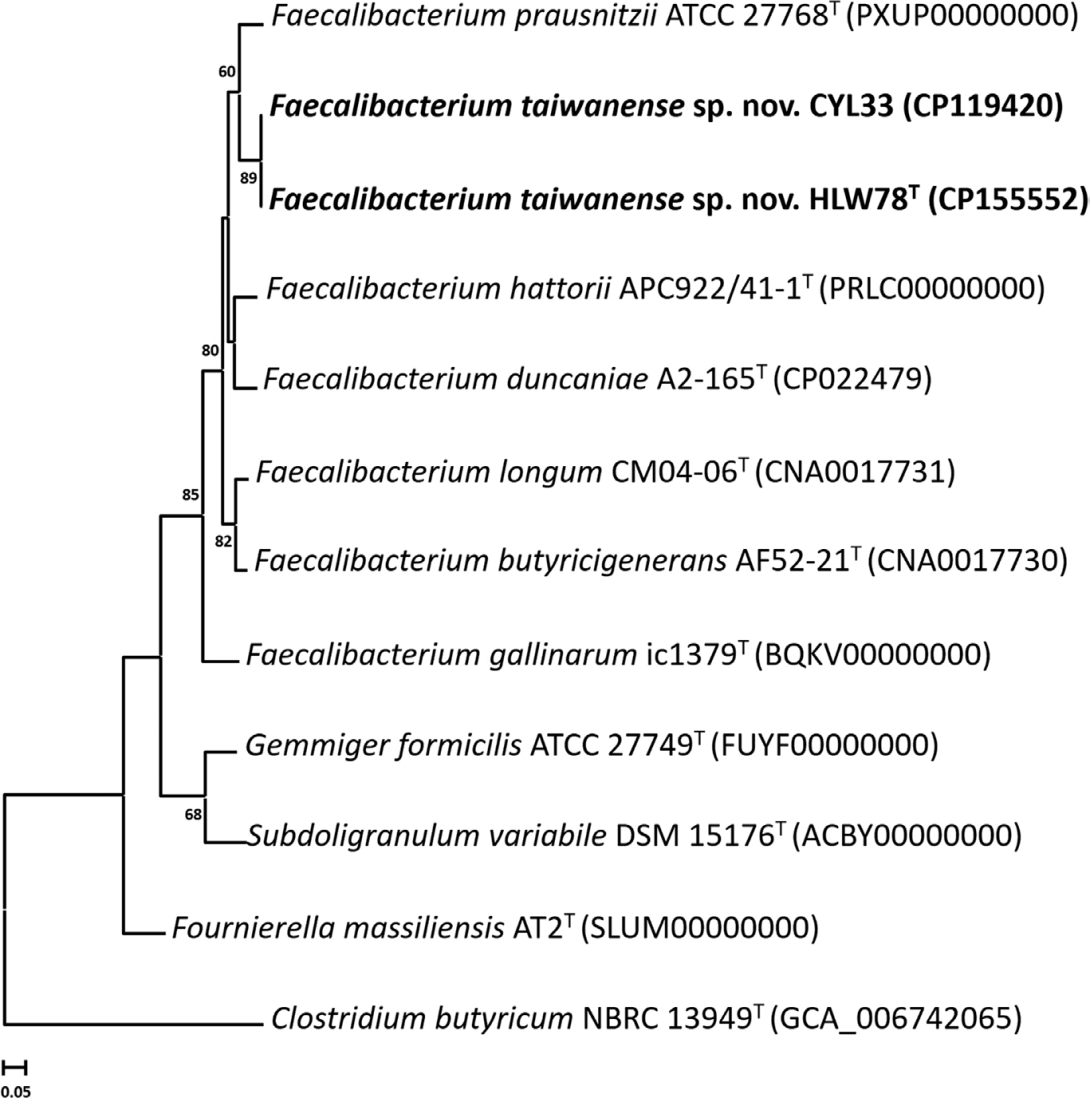
UBCG tree based on 92 bacterial core genes of *Faecalibacterium taiwanense* sp. nov. and the type strains of closely related species. Bootstrap values greater than 60 % are shown at each node and *Clostridium butyricum* NBRC 13949^T^ was used as an outgroup. Bar, 0.05 sequence divergence.

The genome sizes of both strains HLW78^T^ and CYL33 were approximately 2.95 Mbp, as indicated in Table S1, together with their other genomic features. The completeness and contamination levels of the genomic data for strains HLW78^T^ and CYL33 showed high completeness (> 90 %) and low contamination (< 5 %), respectively. This indicates that the genome sequences obtained were suitable for subsequent analysis. The two novel strains HLW78^T^ and CYL33 showed 97.9 % ANI, 96.3 % AAI, and 80.5 % dDDH values, indicating that these two strains are members of the same species. The ANI, AAI and dDDH values of HLW78^T^ with the type strains of six *Faecalibacterium* species were 77.6‒85.1, 71.4‒85.2 and 28.3‒30.9 %, respectively, which were clearly below the cut-off thresholds of ANI (95‒96 %), AAI (95‒96 %) and dDDH (70 %) for delineation of prokaryotic species (Table 1). The phylogenomic trees based on the 92 bacterial core genes, ANI values, and whole genome sequences showed that the novel strains (HLW78^T^ and CYL33) formed an independent cluster and were most closely related to *F. prausnitzii* ATCC 27768^T^ (Figs 3, S2 and S3). These results were consistent with the results of the heatmap and NJ dendrogram analysis (Fig. S4). These analysis results confirmed that strains HLW78^T^ and CYL33 represent a novel species in the genus *Faecalibacterium*. Tanno *et al*. [55] reported that the 88 *Faecalibacterium* strains derived from human faeces were divided into nine distinct groups by ANI-based clustering. Our novel strains were confirmed to have significant high ANI values (96.2‒97.3 %) with the five strains [56] belonging to the Group 3, which are commonly isolated from human faeces and have not yet been classified (Fig. S5). The OrthoVenn2 alignment results indicated that a total of 3009 protein clusters existed within the four *Faecalibacterium* species, of which 1598 were shared by all species. The novel strains HLW78^T^ and CYL33 had 231 unique protein clusters that were not found in any other *Faecalibacterium* species. Interestingly, 67 % of these protein clusters did not exist in either a Swiss protein ID or a gene ortholog annotation (Fig. S6 and Table S2). A total of 2446 and 2417 genes from strains HLW78^T^ and CYL33, respectively, were assigned to 21 COG functional categories (Fig. S7). The most common categories among them belonged to the K (transcription; 242 and 236 genes) and L (recombination and repair; 249 and 237 genes) categories. Among the identified CAZy families, both strains HLW78^T^ and CYL33 possessed a combination of five carbohydrate-binding modules, 21 and 22 glycoside hydrolases, 23 and 22 glycosyl transferases, and four and two polysaccharide lyases, respectively. Furthermore, compared with those in the KnownClusterBlast database [38], the HLW78^T^ and CYL33 genes were found to have three metabolic gene clusters (MGCs) that exhibit greater similarity to reference MGCs. These clusters included the Rnf complex (83 % identical), pathways for converting acetate to butyrate (100 % identical) and pathways for converting putrescine to spermidine (100 % identical). Four *cfrC*, *ermB*, *lnu* (A), *tetW* and one (*ermB*) antimicrobial-resistance genes were identified in the strains HLW78^T^ and CYL33, respectively. No virulence factors and pathogen-associated genes were found.

**Table 1.** Average nucleotide identity (ANI), amino acid identity (AAI) and digital DNA–DNA hybridization (dDDH) values (%) between *Faecalibacterium taiwanense* sp. nov. and its phylogenetically closely related species

Species	Strain	1	2	3	4	5	6	7	8
1, *F. taiwanense* sp. nov.	HLW78^T^	100	80.5	30.9	28.5	29.3	28.3	29.0	22.8
2, *F. taiwanense* sp. nov.	CYL33	97.9*/96.3^†^	100	31.0	28.4	29.6	28.5	29.7	23.0
3, *F. prausnitzii*	ATCC 27768^T^	85.6/85.2	85.2/85.2	100	28.5	28.6	28.4	29.4	22.8
4, *F. hattorii*	APC922/41-1^T^	83.7/82.1	83.6/85.6	86.1/81.9	100	29.8	28.4	29.4	22.6
5, *F. duncaniae*	A2-165^T^	83.9/81.7	84.0/81.8	86.1/80.6	86.0/85.7	100	28.8	28.3	23.1
6, *F. longum*	CM04-06^T^	83.1/80.8	83.3/80.7	85.7/80.4	85.4/82.4	85.6/81.5	100	41.8	23.9
7, *F. butyricigenerans*	AF52-21^T^	83.5/81.8	83.7/82.3	86.1/82.1	86.2/83.6	85.4/81.3	90.2/91.4	100	23.3
8, *F. gallinarum*	ic1379^T^	77.6/71.4	77.7/71.5	81.1/71.2	81.5/71.3	81.8/71.3	82.3/70.9	81.7/71.7	100

The values on the upper right are the dDDH values (%), and the values on the lower left are the *ANI and †AAI values (%).

## Physiology and chemotaxonomy

For morphological, culture and biochemical testing, according to standard techniques, the strains were incubated anaerobically on sterilized BHIS agar at 37 °C for 24 h, unless otherwise stated. The cell morphology of the HLW78^T^ strain was observed through phase-contrast microscopy (Nikon Eclipse E600) and scanning electron microscopy (JSM-6060, jeol). Gram staining was conducted using a Gram-staining kit (Difco) according to the manufacturer’s instructions. A scanning electron microscopy image of cells of strain HLW78^T^ is shown in Fig. S8. Catalase activity was determined by adding 3 % hydrogen peroxide to fresh cells and the oxidase reaction was tested using an oxidase reagent (bioMérieux). Standard methods [57] were followed to determine growth at various temperatures (30–45 °C) and pH values (3.0–10) and in the presence of NaCl and bile. Carbohydrate fermentation and enzymatic activities were determined using the AN MicroPlate system (Biolog), API ZYM and API rapid ID 32A systems (bioMérieux) according to the manufacturers’ instructions. MALDI-TOF MS was performed for whole-cell protein analysis in accordance with a method described elsewhere [22].Whole-cell fatty acids were analysed as fatty acid methyl esters with the Sherlock Microbial Identification System (midi, Inc.) as described previously [58]. The concentrations of organic acids as fermentative end products produced form 25 mM d-glucose in the BHIS medium were determined using an HPLC (model HPLC2695; Waters) equipped a conductive detector (model 432; Waters) and a TSKgel OApak-A column (Tosoh). Analytical conditions were as follows: eluent, 0.75 mM H_2_SO_4_; flow rate, 1 ml min^−1^; and column temperature, 40 °C. Cultivation was performed in triplicate at 37 °C, with analysis of cultures on days 0 and 7.

Various phenotypic features that distinguish the novel species from phylogenetically related species are listed in Table 2. Novel species, comprising strains HLW78^T^ and CYL33, could be differentiated from the closest phylogenetic neighbours, by several features: (1) its ability to ferment mannose and raffinose; (2) its inactivity for *α*-glucosidase (except *F. longum*), leucyl glycine arylamidase and glycine arylamidase.

**Table 2.** Differential phenotypic characteristics of *Faecalibacterium taiwanense* sp. nov. and its phylogenetically closely related species Strains: 1, *F. taiwanense* sp. nov. HLW78^T^; 2, *F. taiwanense* sp. nov. CYL33; 3, *F. butyricigenerans* JCM 39212^T^; 4, *F. duncaniae* JCM 31915^T^; 5, *F. hattorii* JCM 39210^T^; 6, *F. longum* JCM 39208^T^; 7, *F. prausnitzii* BCRC 81047^T^. +, Positive; –, negative; w, weak; nd, no data available.

Characteristic	1	2	3*	4*	5*	6*	7
Temperature range for growth (°C)	30–45	30–45	30–42	30–42	30–42	30–42	30–42
pH range for growth	5–9	5–9	6–7.5	5.5–7.5	5.5–7.5	5.5–7.5	5.5–7.5
NaCl concentration for growth (%)	0–0.5	0–0.5	0–1	0–1	0–1	0–3	0–3
API ZYM results:							
Leucine arylamidase	+	+	nd	‒	w	nd	+
Acid phosphatase	+	+	nd	w	w	nd	+
*β*-Glucuronidase	‒	+	w	+	w	w	+
*β*-Glucosidase	+	+	w	‒	w	w	+
API Rapid ID 32A results:							
6-Phospho-*β*-galactosidase	‒	+	‒	+	+	‒	‒
*α*-Glucosidase	‒	‒	w	+	+	‒	+
*β*-Glucosidase	+	+	‒	‒	+	+	‒
*β*-Glucuronidase	‒	‒	‒	+	+	‒	‒
Mannose fermentation	+	+	‒	‒	‒	‒	+
Raffinose fermentation	+	+	‒	‒	‒	‒	+
Leucyl glycine arylamidase	‒	‒	w	+	+	w	+
Phenylalanine arylamidase	+	+	nd	‒	‒	nd	+
Leucine arylamidase	+	+	nd	‒	‒	nd	+
Tyrosine arylamidase	+	+	nd	‒	‒	nd	+
Alanine arylamidase	+	+	nd	‒	‒	nd	+
Glycine arylamidase	‒	‒	w	+	+	w	+
Serine arylamidase	+	+	nd	‒	‒	nd	+
AN MicroPlate results:							
d-Gluconic acid	‒	‒	nd	nd	nd	nd	+
Palatinose	+	‒	nd	nd	nd	nd	+
Glyoxylic acid	‒	‒	nd	nd	nd	nd	+
*α-*Hydroxybutyric acid	‒	‒	nd	nd	nd	nd	+
*α*-Ketobutyric acid	‒	+	nd	nd	nd	nd	‒
d,l-Lactic acid	‒	‒	nd	nd	nd	nd	+
l-Lactic acid	‒	‒	nd	nd	nd	nd	+
d-Lactic acid methyl ester	‒	+	nd	nd	nd	nd	+
d-Malic acid	‒	‒	nd	nd	nd	nd	+
Pyruvic acid	‒	+	nd	nd	nd	nd	‒
d-Saccharic acid	‒	‒	nd	nd	nd	nd	+
*meso*-Tartaric acid	‒	‒	nd	nd	nd	nd	+

*Data for *F. butyricigenerans*, *F. duncaniae*, *F. hattorii* and *F. longum* from Sakamoto *et al*. [17] and Zhou *et al.* [16].

The comparison of MALDI-TOF MS spectra of strains HLW78^T^ and CYL33 with *F. prausnitzii* BCRC 81047^T^ revealed six specific peaks at 2989, 3134, 3183, 4487, 4855 and 6271* m/z* that discriminated strains HLW78^T^ and CYL33 from *F. prausnitzii* BCRC 81047^T^ (Fig. S9). The cellular fatty acid compositions of the novel strains were similar, containing C_16 : 0_ (30.5–30.9 %), C_18 : 1_* ω*7*c* (10.6–14.5 %), and C_18 : 1_* ω*7*c* DMA (10.8–12.1 %), as major fatty acids (>10 % of total). The ratio of C_16:0,_ C_16 : 0_ DMA and C_18 : 1_* ω*7*c* DM present in novel strains were higher than that of other strains, and could be clearly differentiated from closest *Faecalibacterium* species based on their cellular fatty acid compositions, which contained C_18 : 1_* ω*7*c* and lacked C_15 : 0_ and C_17 : 1_* ω*6*c*, respectively (Table 3). The major metabolic end-product of strains HLW78^T^ and CYL33 was butyric acid, with acetate consumption identified as the main driver of butyrate production by *F. prausnitzii* [59].

**Table 3.** Cellular fatty acid compositions (%) of *Faecalibacterium taiwanense* sp. nov. and its phylogenetically closely related species Strains: 1, *F. taiwanense* sp. nov. HLW78^T^; 2, *F. taiwanense* sp. nov. CYL33; 3, *F. butyricigenerans* JCM 39212^T^; 4, *F. duncaniae* JCM 31915^T^; 5, *F. hattorii* JCM 39210^T^; 6, *F. longum* JCM 39208^T^; 7, *F. prausnitzii* BCRC 81047^T^. Data (except for *F. butyricigenerans*, *F. duncaniae*, *F. hattorii* and *F. longum* from Sakamoto *et al.* [17]) were obtained in this study. Values are percentages of total fatty acids. Fatty acids present at >10 % are indicated in bold. ‒, Not detected.

Fatty acid	1	2	3	4	5	6	7
C_12 : 0_	‒	‒	2	2.6	‒	‒	2.7±0.4
C_13 : 0_	‒	‒	2.5	‒	‒	‒	2.5
C_14 : 0_	8.7±0.4	4.8±0.1	8.1±3.3	5.3±1.3	4.9±1.7	6.3±1.0	**10.4±5.2**
C_15 : 0_	‒	‒	**26.2±8.5**	**13.8±2.1**	**16.8±2.5**	**19.6±3.0**	**16.3±2.1**
C_16 : 0_	**30.5±0.7**	**39.1±0.5**	**17.3±6.5**	**14.2±2.5**	**21.2±4.0**	**16.5±2.8**	**10.8±2.4**
C_17.:0_	‒	‒	6.6±3.2	3.2±1.4	3.1±0.8	2.2	3.1±0.8
C_15 : 1_* ω*6*c/*t8	‒	‒	‒	1.5	‒	1.5±0.6	‒
C_16 : 1_* ω*5*c*	2.2±0.1	1.4	‒	‒	‒	‒	‒
C_16 : 1_* ω*7*c*	**11.7±0.2**	6.3±0.1	8.1±3.3	7.8±1.4	7.6±1.8	7.4±1.7	5.6±1
C_16 : 1_* ω*9*c*	4.7±0.1	2.7±0.1	‒	2.8±0.2	‒	2.2	2.5
C_17 : 1_* ω*6*c*	‒	‒	9.1±4.0	**11.8±2.3**	5.9±0.3	6.8±1.9	6.7±2.5
C_14 : 0_ DMA	1.8±0.1	1.1±0.1	2.1	2	2.4	1.8	6.9
C_18 : 1_* ω*7*c*	**10.6±0.3**	**14.5±0.3**	‒	‒	‒	‒	‒
C_16 : 0_ ALDE	2.0±0 .1	1.9±0.2	2.3	1.1	2.3±0.3	1.5±0.3	‒
C_16 : 0_ DMA	8.8±0.2	9.1±0.3	7.2±0.8	6.6	7.7±1.2	6.9±1.2	5.5±0.7
C_16 : 1_* ω*7*c* DMA	‒	‒	2.2	4.3±0.7	3.1±0.4	3.0±1.6	3.2±1.3
C_16 : 1_* ω*9*c* DMA	3.3±0.2	1.8±0.1	‒	‒	‒	‒	‒
C_17 : 0_ DMA	‒	‒	5.3±1.9	‒	‒	1.8	2.4±0.4
C_18 : 1_* ω*7*c* DMA	**10.8±0.1**	**12.1±0.2**	5.1	7.9±1.1	**10.7±3.5**	6.6±0.9	4.7±1.5
C_18 : 1_ at 17.254 DMA	‒	‒	3.6±1.3	3.4±0.7	3.5±0.2	3.1±0.1	3.5±0.5
iso-C_15 : 0_	‒	‒	3.5±1.9	2.2±0.1	1.7	3.0±0.5	2.4±0.4
iso-C_17 : 0_	‒	‒	‒	1.3	‒	‒	1.9
Summed feature 1*	‒	‒	1.3	‒	1.5	‒	2.3±0.6
Summed feature 2*	‒	‒	‒	‒	‒	‒	1.4
Summed feature 4*	‒	‒	2.5	3.6±0.4	3.6	3.7±0.9	2.9±0.7
Summed feature 5*	‒	‒	5.2±0.5	2.5±0.2	3.7±0.6	3.6±0.7	6.6±0.8
Summed feature 6*	‒	‒	‒	1.3	‒	‒	‒
Summed feature 8*	‒	‒	2.6	5.6±1.5	4.6±0.5	3.1±0.5	3.4±1.4
Summed feature 10*	‒	‒	2.5	6.0±1.4	3.9±1.1	4.6±1.2	4.2

*Summed features are fatty acids that cannot be resolved reliably from another fatty acid using the chromatographic conditions chosen. The midi system groups these fatty acids together as one feature with a single percentage of the total. Summed feature compositions: 1, C_13 : 1_* ω*1*c* and/or C_14 : 0_ ALDE; 2, C_12 : 0_ 3OH and/or C_13 : 0_ DMA; 4, unknown 14.762 and/or C_15 : 2_; 5, C_15 : 0_ DMA and/or C_14 : 0_ 3OH; 6, anteiso-C_15 : 0_ 3OH and/or C_16 : 1_* ω*9*c* DMA; 8, C_17 : 1_* ω*8*c* and/or C_17 : 2_; 10, C_18 : 1_ c11/t9/t6 and/or unknown 17.834.Data for , , and from Sakamoto [].

Accordingly, the results obtained in the phenotypic characterization, genomic and phylogenetic analysis and chemotaxonomic analyses demonstrated that the novel strains HLW78^T^ and CYL33 represented a single, novel species within the genus *Faecalibacterium*, for which the name *Faecalibacterium taiwanense* sp. nov. is proposed, with strain HLW78^T^ (=BCRC 81397^T^= NBRC 116372^T^) as the type strain.

## Description of *Faecalibacterium taiwanense* sp. nov.

*Faecalibacterium taiwanense* (tai.wan.en′se. N.L. neut. adj. *taiwanense*, pertaining to Taiwan, where the type strain was isolated).

Cells are Gram-stain-negative, non-spore-forming, non-motile rods (0.5‒1.2×2.5‒14.5 µm) and strictly anaerobic. Cells are negative for reduction of nitrates, indole, catalase and urease production. Colonies are approximately 0.5–1.5 mm in diameter, yellowish, circular, entire and slightly convex after 2 days at 37 °C on BHIS agar plates under anaerobic conditions. Growth occurs between 30–45 °C and pH 5–9, and tolerates 0–0.5 % (w/v) NaCl. The type strain is not able to grow in the presence of 2 % oxgall (w/v). Aesculin is hydrolysed, but gelatin is not. Positive reactions are obtained using API ZYM system for alkaline phosphatase, leucine arylamidase, acid phosphatase, *α*-glucosidase and *β*-glucosidase. Negative reactions for esterase, esterase lipase, lipase, valine arylamidase, cystine arylamidase, trypsin, *α*-chymotrypsin, *α*-galactosidase, *N*-acetyl-*β*-glucosaminidase, *α*-mannosidase and *α*-fucosidase. *β*-Glucuronidase is variable. In Rapid ID 32A strips, positive reactions are obtained from *β*-galactosidase, alkaline phosphatase, arginine dihydrolase, phenylalanine arylamidase, leucine arylamidase, tyrosine arylamidase, alanine arylamidase, histidine arylamidase and glutamyl glutamic acid arylamidase. Negative reactions are obtained from urease, arginine dihydrolase, α-galactosidase, α-arabinosidase, *β*-glucuronidase, *N*-acetyl-*β*-glucosaminidase, glutamic acid decarboxylase, α-fucosidase, proline arylamidase, leucyl glycine arylamidase, pyroglutamic arylamidase, glycine arylamidase and glutamyl glutamic acid arylamidase. *β*-Galactosidase-6-phosphate and *β*-glucosidase are variable. Mannose and raffinose are fermented. Using the AN MicroPlate system, positive reaction is obtained from pyruvic acid methyl ester. Palatinose, d-lactic acid methyl ester, pyruvic acid and l-serine are variable. The major cellular fatty acids and metabolic end-product are C_16 : 0_, C_18 : 1_* ω*7*c* and C_18 : 1_* ω*7*c* DMA and butyric acid, respectively. The type strain is HLW78^T^ (=BCRC 81397^T^=NBRC 116372^T^), which was isolated from faeces of a healthy Taiwanese adult. The genomic DNA G+C content of the type strain is 55.9 mol%. The GenBank/EMBL/DDBJ accession numbers are OQ749743 (16S rRNA gene), OQ784576 (*mutL* gene) and CP155552 (complete genome), respectively.

## supplementary material

10.1099/ijsem.0.006413 Uncited Supplementary Material 1.

10.1099/ijsem.0.006413 Uncited Supplementary Material 2.

## Abbreviations

AAI: amino acid identity
ANI: average nucleotide identity
BHIS: brain heart infusion supplemented
dDDH: digital DNA–DNA hybridization
ME: minimum-evolution
MGC: metabolic gene cluster
ML: maximum-likelihood
NJ: neighbour-joining
