# Supplementary material for: Faecalibacterium taiwanense sp. nov., isolated from human faeces
Source: Int J Syst Evol Microbiol. 2024 Jun 7;74(6):006413. doi: 10.1099/ijsem.0.006413 (PMC11261667; doi:10.1099/ijsem.0.006413)
Supplement: Uncited Supplementary Material 1. [file ijsem-74-06413-s001.pdf]

IJSEM Supplementary material for:

Faecalibacterium taiwanense **sp. nov., isolated from human faeces**

Jong-Shian Liou[1], Wei-Ling Zhang[1], Li-Wen Hsu[1], Chih-Chieh Chen[2,3], Yu-Ting Wang[4], Koji Mori[5], Kohei Hidaka[5], Moriyuki Hamada[5], Lina Huang[1], Koichi Watanabe[6*], Chien-Hsun Huang[1*]

Author affiliations:

[1] Bioresource Collection and Research Center, Food Industry Research and Development Institute, 331 Shih-Pin Rd, Hsinchu 30062, Taiwan, ROC

[2] Institute of Medical Science and Technology, National Sun Yat-sen University, Kaohsiung 80424, Taiwan, ROC

[3] Rapid Screening Research Center for Toxicology and Biomedicine, National Sun Yat-sen University, Kaohsiung 80424, Taiwan, ROC

[4] Division of Research and Analysis, Food and Drug Administration, Ministry of Health and Welfare, Taipei 11561, Taiwan, ROC

[5] Biological Resource Center, National Institute of Technology and Evaluation (NBRC), 2-5-8 Kazusakamatari, Kisarazu, Chiba 292-0818, Japan

[6] Department of Animal Science and Technology, National Taiwan University, No. 50, Lane 155, Sec 3, Keelung Rd., Taipei 10673, Taiwan, ROC

*Corresponding author

Koichi Watanabe
E-mail: koichi_wtnb@yahoo.co.jp

Chien-Hsun Huang
E-mail: chh@firdi.org.tw

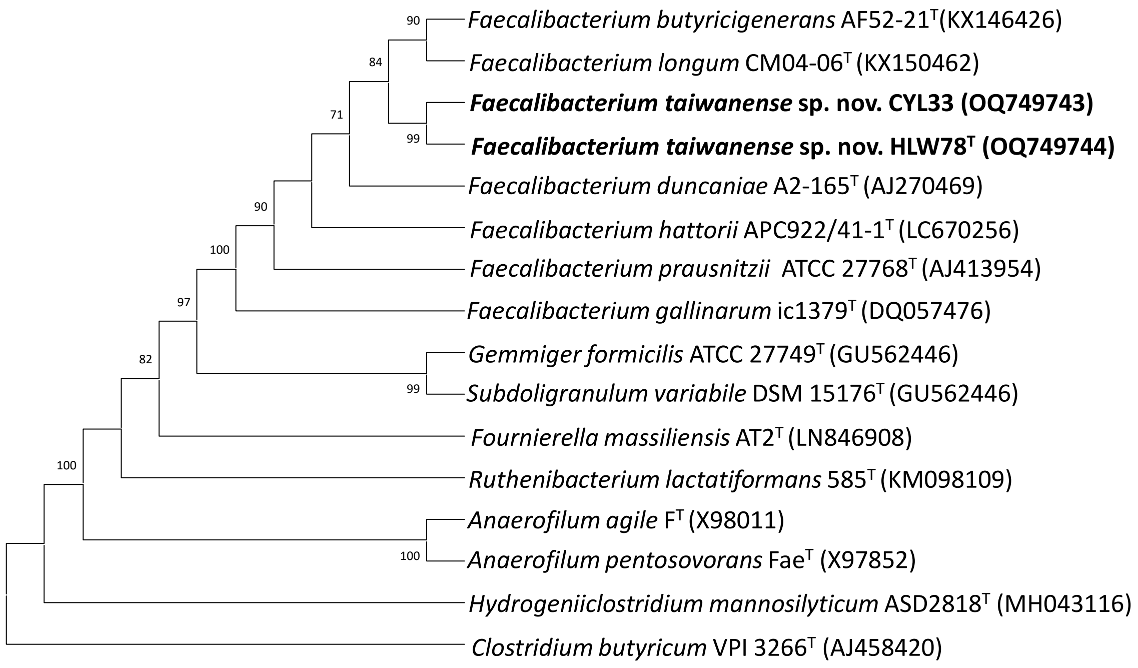

Supplementary Fig. S1. Phylogenetic tree based on 16S rRNA gene sequences showing the relationship of *Faecalibacterium taiwanense* sp. nov. with the type strains of closely related species. The tree was constructed by maximum parsimony method based on a comparison of approximately 1373 bp, and *Clostridium butyricum* VPI 3266^T was used as an outgroup. Bootstrap values (>70%) based on 1000 replicates are shown at branch nodes.

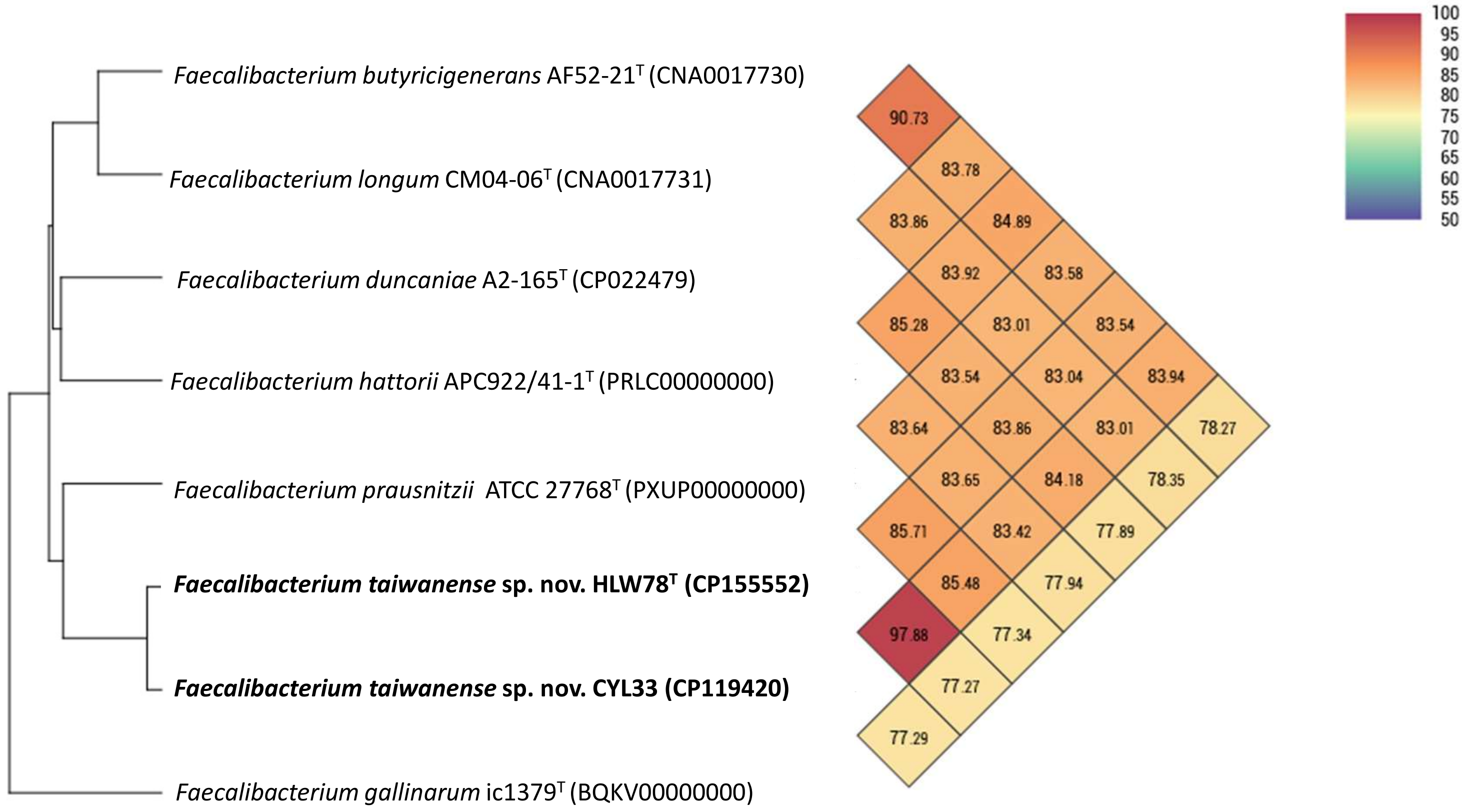

Supplementary Fig. S2. OrthoANI values between *Faecalibacterium taiwanense* sp. nov. and its phylogenetically related species.

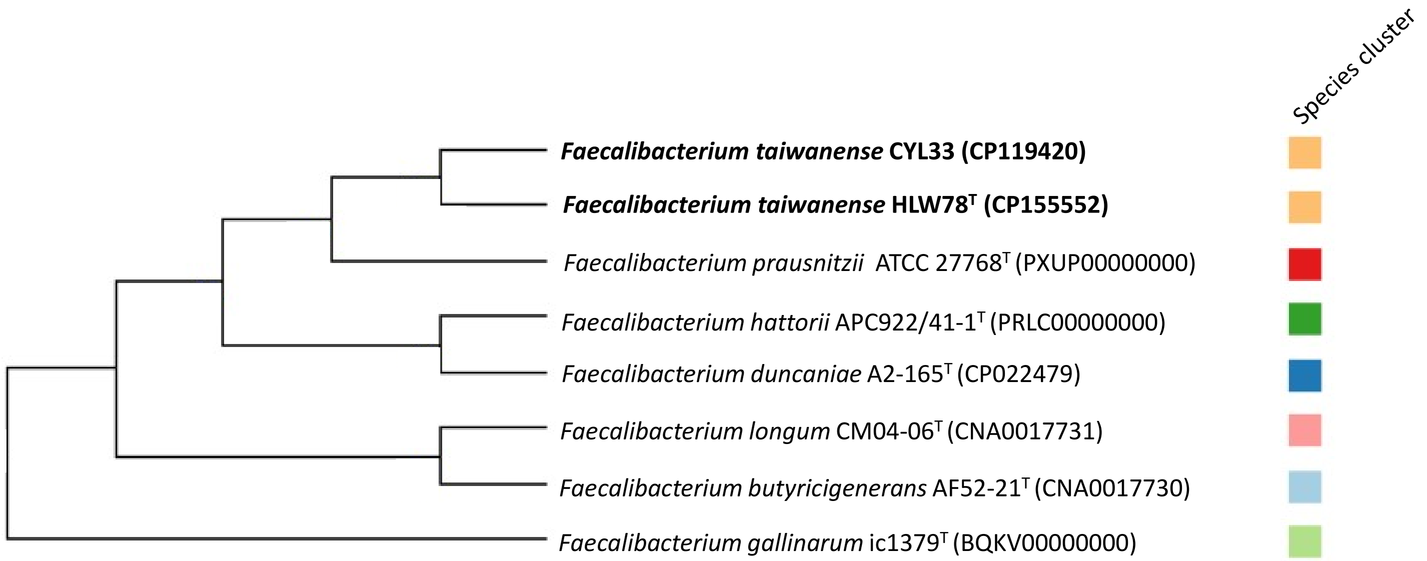

Supplementary Fig. S3. Phylogenomic tree based on TYGS results showing the relationship between *Faecalibacterium taiwanense* sp. nov. and its phylogenetically related species. The tree was inferred with FastME 2.1.6.1 from GBDP distances calculated from genome sequences. The branch lengths are scaled in terms of GBDP distance formula d_5. The tree was rooted at the midpoint.

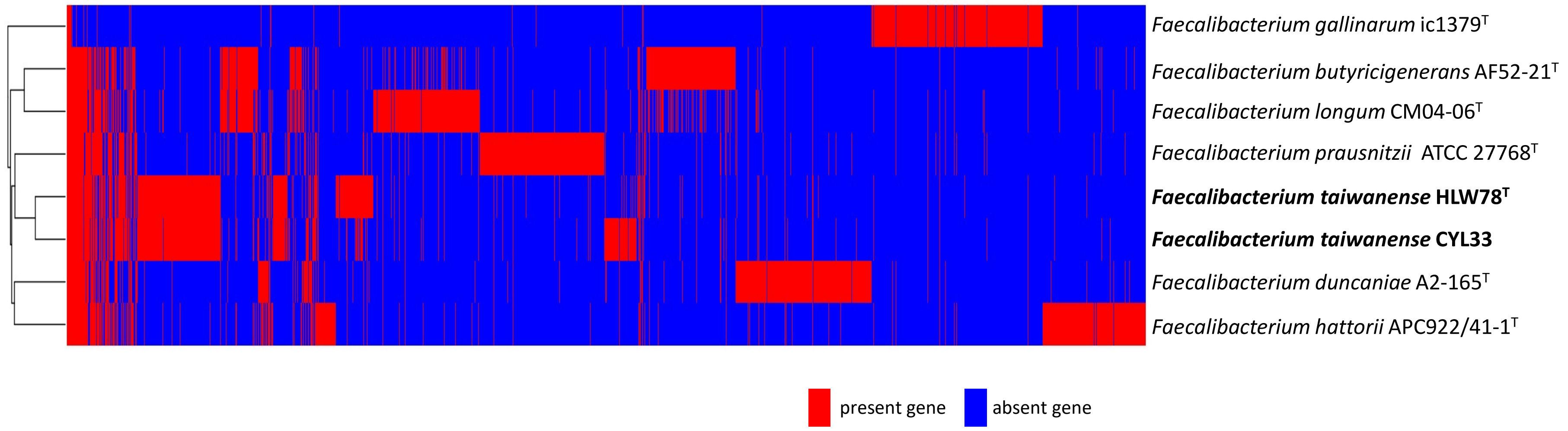

Supplementary Fig. S4. Heatmap and NJ dendrogram of the analyzed eight *Faecalibacterium* strains based on the presence or absence of genes. Accession numbers of the genome sequences used for the reconstruction are shown in Supplementary Table S1.

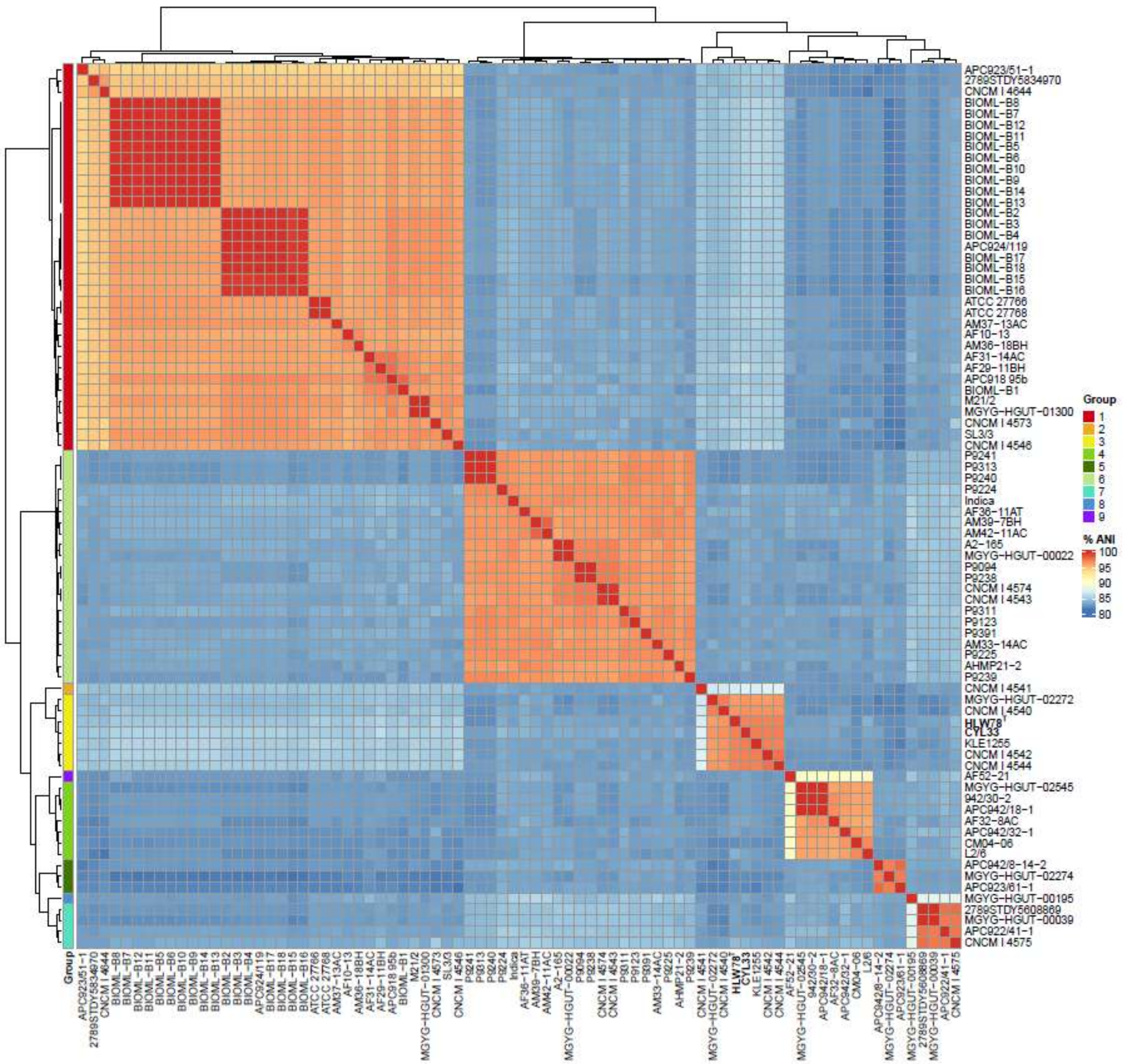

Supplementary Fig. S5. Heatmap and hierarchical clustering of the analyzed 80 *Faecalibacterium* strains based on the ANI values.

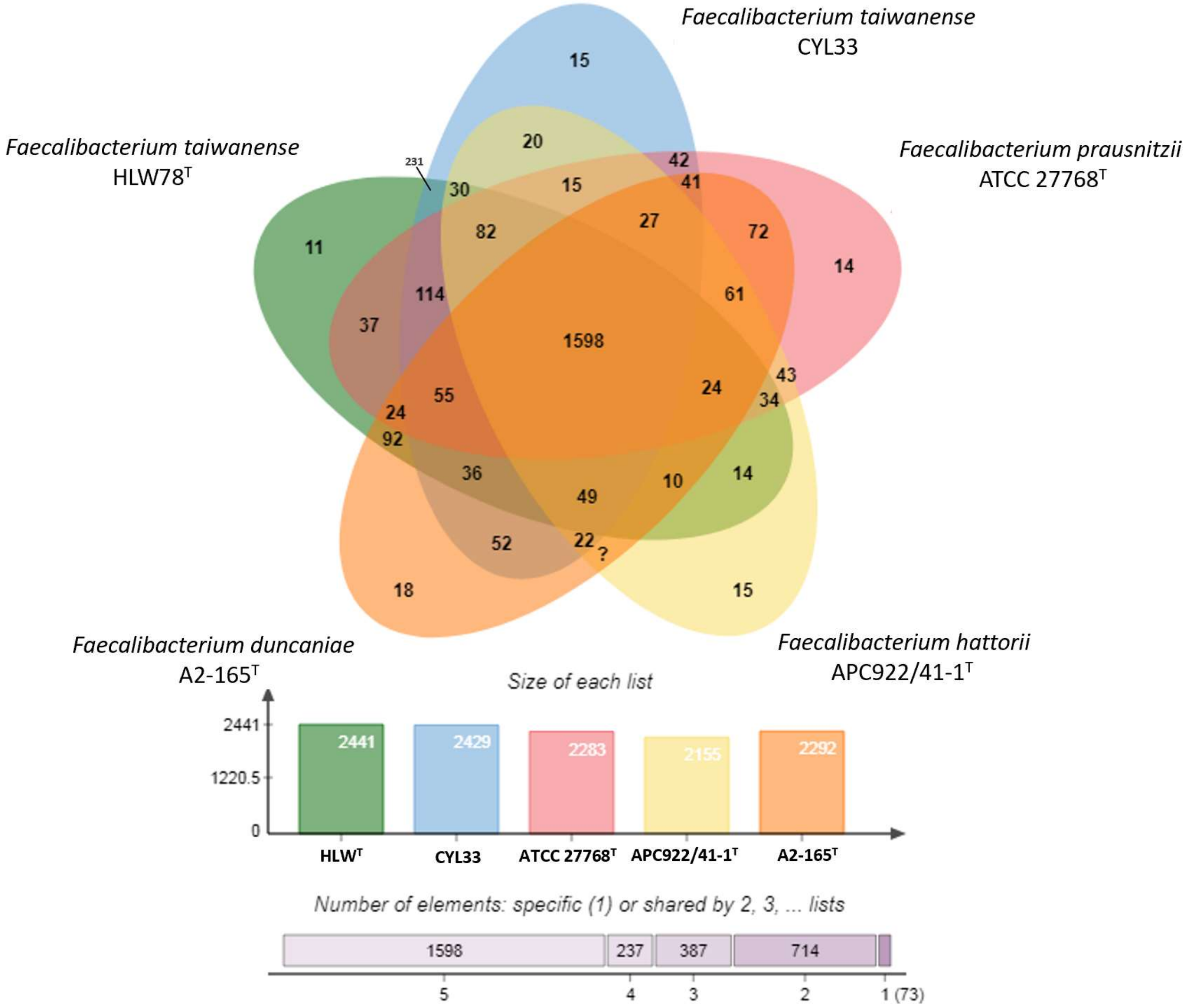

Supplementary Fig. S6. The Venn diagram and bar plots generated by OrthoVenn2 illustrate the distribution of shared and unique gene clusters among different *Faecalibacterium* species.

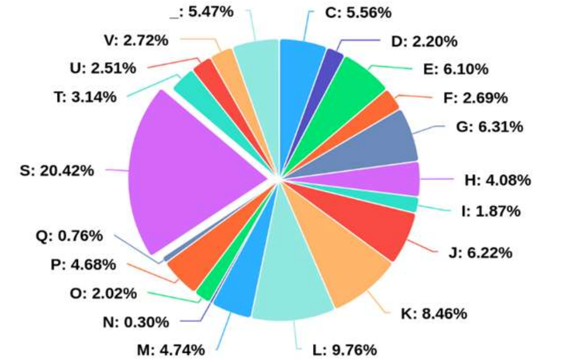

***Faecalibacterium taiwanense* HLW78^T**

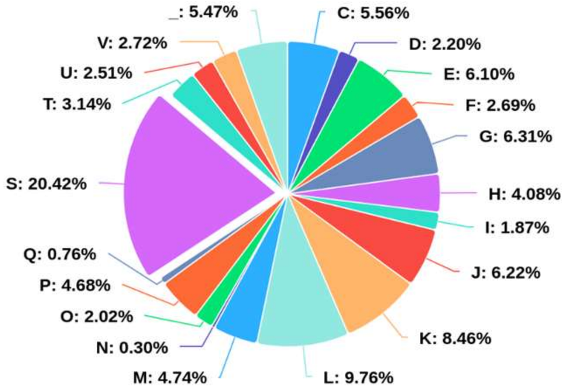

***Faecalibacterium taiwanense* CYL33**

A：RNA processing and modification
B：Chromatin structure and dynamics
C：Chromatin structure and dynamics
D：Cell cycle control, cell division, chromosome partitioning
E：Amino acid transport and metabolism
F：Nucleotide transport and metabolism
G：Carbohydrate transport and metabolism
H：Coenzyme transport and metabolism
I：Lipid transport and metabolism
J：Translation, ribosomal structure and biogenesis
K：Transcription
L：Replication, recombination and repair
M：Cell wall/membrane/envelope biogenesis
N：Cell motility
O：Posttranslational modification, protein turnover, chaperones
P：Inorganic ion transport and metabolism
Q：Secondary metabolites biosynthesis, transport and catabolism
S：Function unknown
T：Signal transduction mechanisms
U：Intracellular trafficking, secretion, and vesicular transport
V：Defense mechanisms
_：Unclassified

Supplementary Fig. S7. Results of an eggNOG functional category analysis of strains HLW78^T and CYL33. The major two parts of 2551 and 2444 COG categories in strains HLW78^T and CYL33, respectively, are K (Transcription) and L (Replication, recombination and repair.

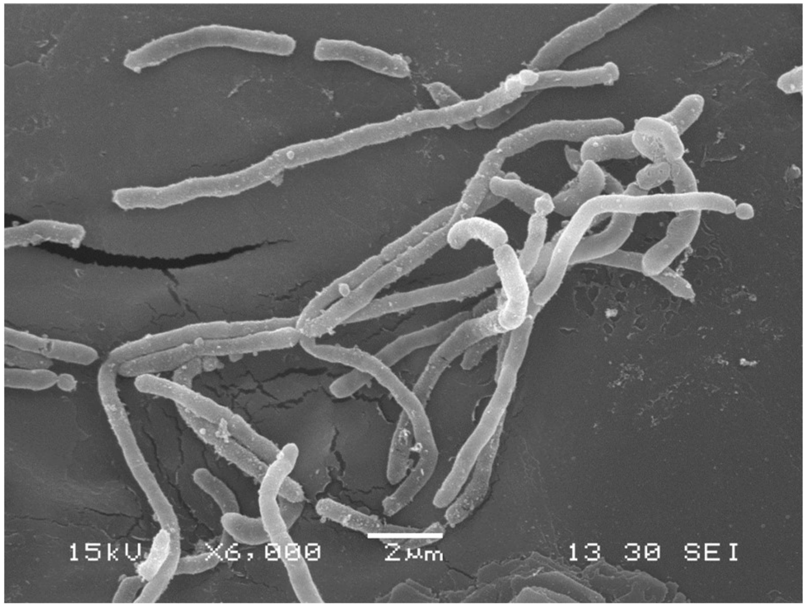

Supplementary Fig. S8. Scanning electron micrograph of strain HLW78^{T} after anaerobic cultivation on modified BHIS agar plates at 37 °C for 2 days.

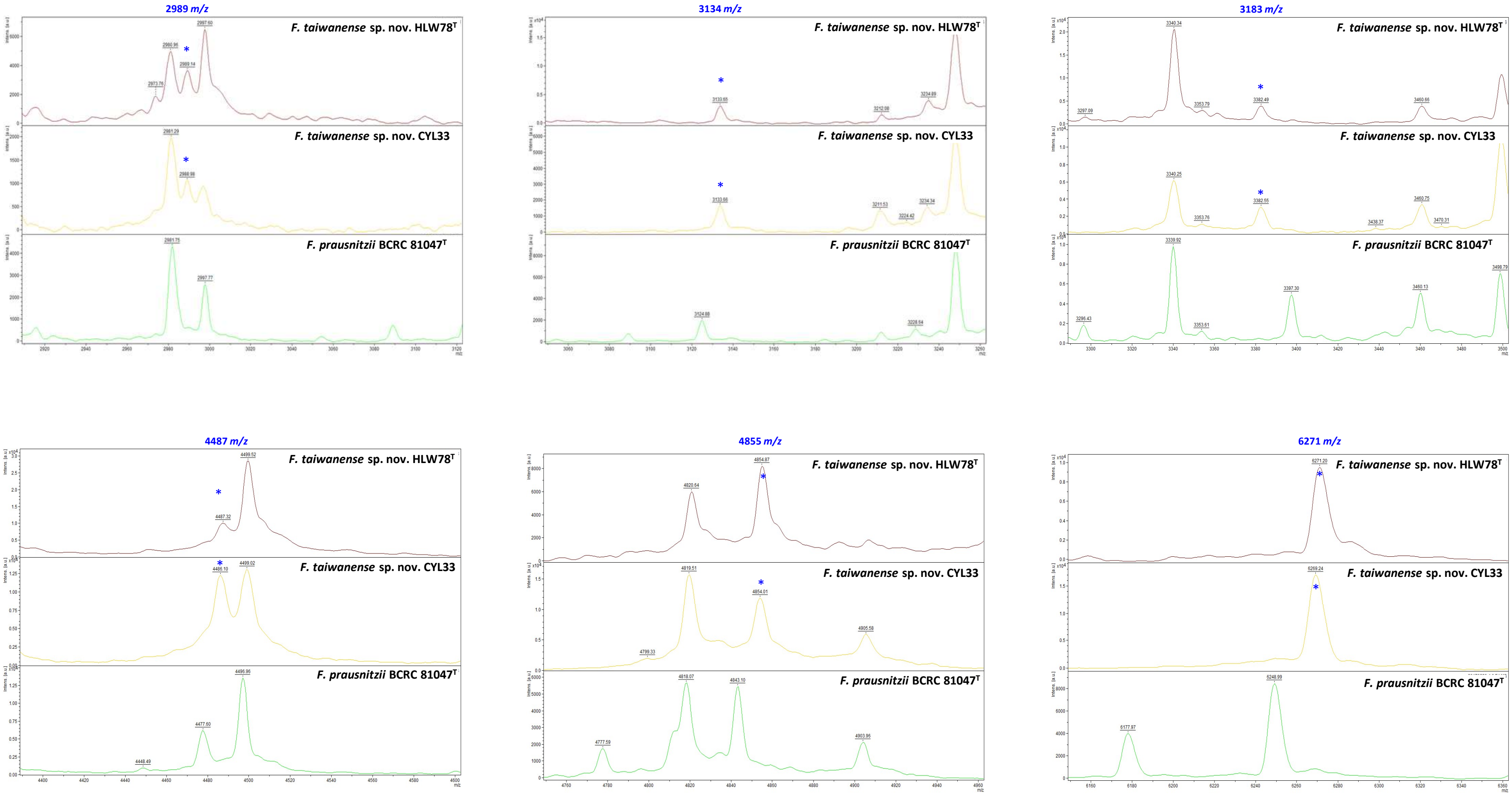

Supplementary Fig. S9. MALDI-TOF MS spectra of cell lysates of *Faecalibacterium taiwanense* sp. nov. strains HLW78^{T} and CYL33 and *F. prausnitzii* BCRC 81047^{T}. The species-specific peaks for the *F. taiwanense* sp. nov. are indicated by asterisks (2989, 3134, 3183, 4487, 4855 and 6271 *m/z*). a.u., arbitrary units; *m/z*, mass-to-charge ratio.
